# Development and application of ubiquitin-based chemical probes†

**DOI:** 10.1039/d0sc03295f

**Published:** 2020-08-04

**Authors:** Xin Sui, Yu Wang, Yun-Xiang Du, Lu-Jun Liang, Qingyun Zheng, Yi-Ming Li, Lei Liu

**Affiliations:** School of Food and Biological Engineering, Key Laboratory of Metabolism and Regulation for Major Diseases of Anhui Higher Education Institutes, Hefei University of Technology Hefei 230009 China ymli@hfut.edu.cn; Tsinghua-Peking Center for Life Sciences, Ministry of Education Key Laboratory of Bioorganic Phosphorus Chemistry and Chemical Biology, Center for Synthetic and Systems Biology, Department of Chemistry, Tsinghua University Beijing 100084 China lliu@mail.tsinghua.edu.cn

## Abstract

Protein ubiquitination regulates almost every process in eukaryotic cells. The study of the many enzymes involved in the ubiquitination system and the development of ubiquitination-associated therapeutics are important areas of current research. Synthetic tools such as ubiquitin-based chemical probes have been making an increasing contribution to deciphering various biochemical components involved in ubiquitin conjugation, recruitment, signaling, and deconjugation. In the present minireview, we summarize the progress of ubiquitin-based chemical probes with an emphasis on their various structures and chemical synthesis. We discuss the utility of the ubiquitin-based chemical probes for discovering and profiling ubiquitin-dependent signaling systems, as well as the monitoring and visualization of ubiquitin-related enzymatic machinery. We also show how the probes can serve to elucidate the molecular mechanism of recognition and catalysis. Collectively, the development and application of ubiquitin-based chemical probes emphasizes the importance and utility of chemical protein synthesis in modern chemical biology.

## Abstract 2

This article reviews the design, synthesis, and application of different classes of Ub-based chemical probes.

## Graphical abstract

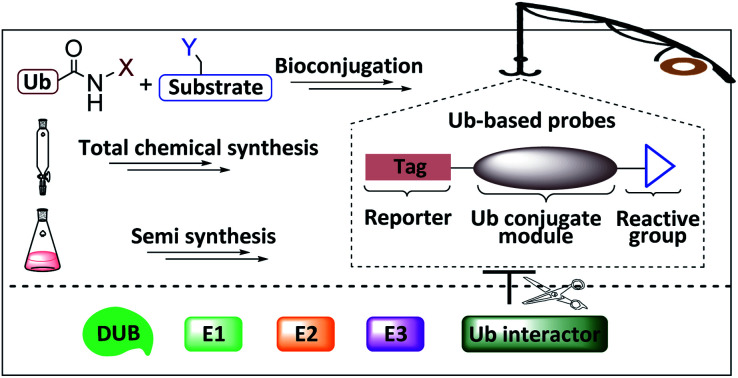

## 1. Introduction

**Fig. 1:**
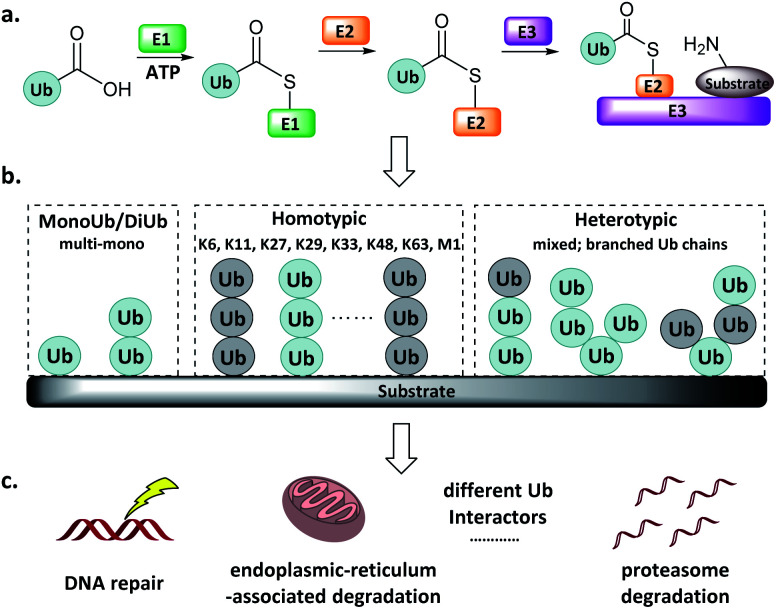
Ub system. (a) Actions of E1, E2, and E3. (b) Mono-, di- and poly-Ub chains. (c) Distinct Ub chains are recognized by different interactors.

Protein post-translational modifications (*e.g.*, methylation, acetylation, phosphorylation, glycosylation, and ubiquitination) regulate various biological processes in all eukaryotic cells, and dysregulation of the associated enzymes gives rise to diverse pathologies. Ubiquitination, the post-translational attachment of a 76-residue protein named ubiquitin (Ub), is orchestrated by the actions of four enzyme classes.^1–3^ The Ub activating enzyme E1 catalyses the formation of an E1-Ub thioester at the expense of ATP (Fig. 1a). Then, the active Cys of the conjugating enzyme E2 attacks the E1-Ub thioester to produce an E2-Ub thioester, and the Ub ligase E3 transfers the Ub from the active Cys of E2 to the Lys of the substrate protein. The reverse of this process is accomplished by deubiquitinating enzymes (DUBs), which catalyse the cleavage of the isopeptide bond.^4^

Ub can be attached to substrate proteins as a single unit or in the form of Ub chains, wherein successive Ubs are connected at Ub's M1, K6, K11, K27, K29, K33, K48, and K63 amino groups. In addition to the homotypic Ub chains, heterotypic and branched Ub chains have also been discovered^5^ (Fig. 1b). These Ubs adopt distinct conformations, which differentially influence the fate of the protein to which they are attached in a manner reminiscent of a code. For example, K48-linked Ubs signal proteasome degradation, while K63-linked Ubs regulate the innate immune signalling pathways.^1–3,6,7^ In addition, Ub-like (UbL) modifications such as Nedd8, SUMO, and ISG15 have also been identified.^8–10^ Defects in components of the Ub/UbL processes influence disease pathogenesis, especially cancer and neurodegeneration. A more detailed understanding of Ub/UbL modifications in cellular processes such as DNA repair and immune response remains to be acquired (Fig. 1c).

Genetic and proteomic methods have been developed to study ubiquitination. For instance, proteomic studies using an antibody targeting Lys-ε-Gly–Gly have revealed >50 000 ubiquitination sites in human cells.^11^ More recently, a Ub clipping method was developed to map Ubs *in vivo*, showing that branched Ubs account for 10–20% of the total Ub abundance.^12^ Despite these advances, understanding of ubiquitination processes is far from complete, and currently available tools are inadequate to fill in the gaps. For example, elucidation of the E2, E3, or DUB responsible for a specific reaction remains difficult using genetic mutations or small interfering RNA; and monitoring of the dynamics of the reversible ubiquitination processes requires higher levels of spatiotemporal resolution.

**Fig. 2:**
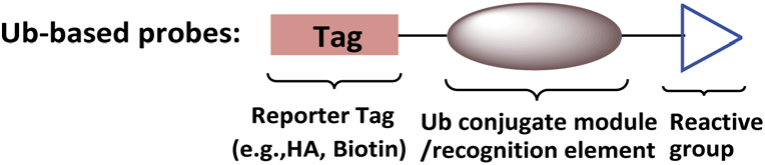
Ub-based chemical probes usually comprise a Ub conjugate module, a reactive group and a reporting group.

To supplement canonical methods for the study of ubiquitination, Ub-based chemical probes have been developed to capture or monitor Ub-related enzymes and interactors either covalently or non-covalently.^13^ Ub-based chemical probes usually comprise a reactive group, a reporting group, and a Ub conjugate module (Fig. 2). The Ub conjugate module contains either a monomeric Ub, Ub chain, or ubiquitinated substrate protein. The reactive group can be used to capture or enrich the Ub enzymes and interactors. The reporting group is used for visualization and/or identification.

Ub-based chemical probes have been demonstrated to be effective tools for discovering and monitoring Ub-related enzymes and interactors.^14–16^ They can also be used to study the mechanism of the ubiquitination or deubiquitination event.^17–19^ Further development of Ub-based chemical probes with expanded functions and enhanced sensitivity is an important area in Ub research. This article aims to review the different classes of Ub-based chemical probes and their applications.

## 2. Designs of Ub-based chemical probes

### 2.1 Probes capturing enzyme active sites

**Fig. 3:**
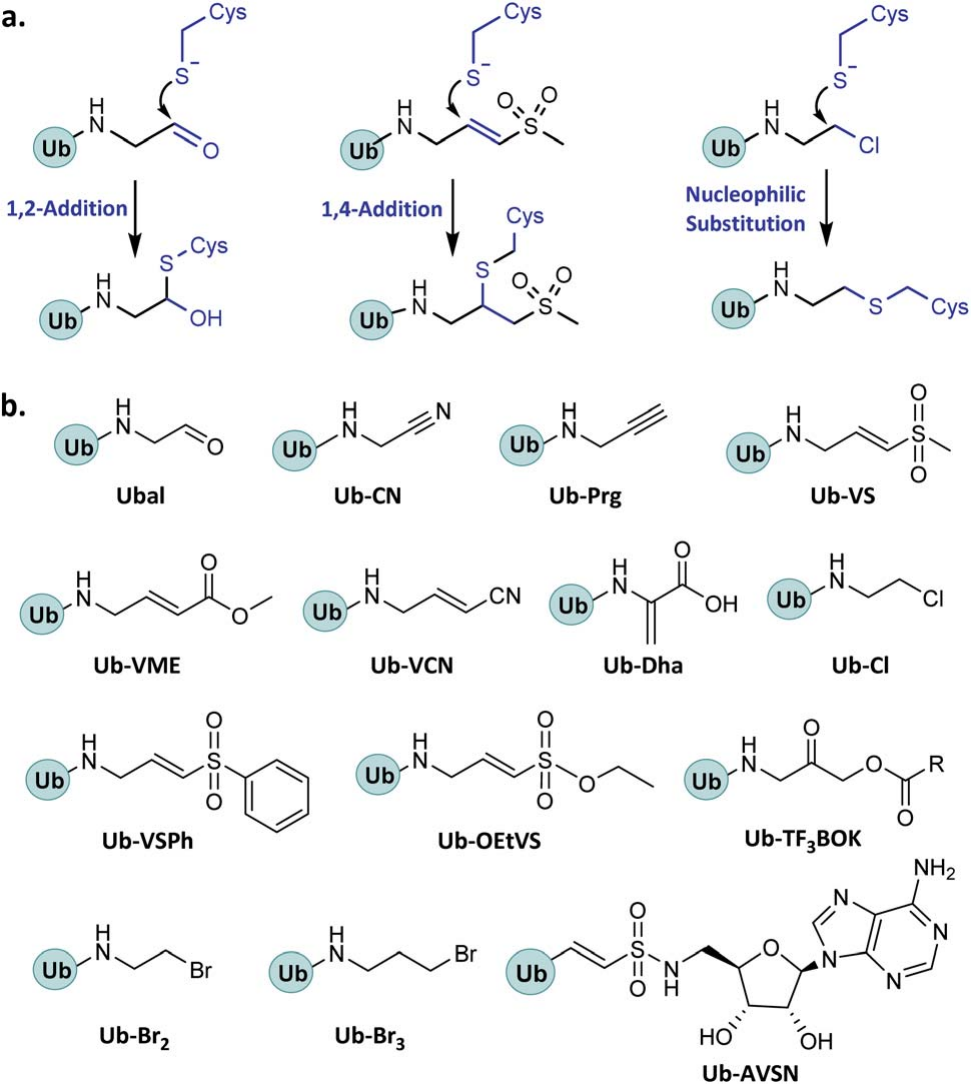
Probes capturing active Cys. (a) General mechanisms. (b) Structures.

In enzymatic ubiquitination and deubiquitination processes, the catalytic site is usually an active Cys. Strategies to capture the active Cys commonly rely on nucleophilic addition or substitution reactions (Fig. 3).

#### 2.1.1 1,2-Addition

The first probes (*i.e.* Ubal and Ub-CN)^14^ developed for capturing DUBs can react with the active Cys through 1,2-addition. Both of these reactions are reversible, the resulting complex usually being unstable under strongly reducing conditions. To solve this problem, the more powerful probe Ub-Prg (also called Ub-PA) was developed.^20,21^ Ub-Prg can react with the active Cys of DUBs through 1,2-addition to produce a stable vinyl thioether product.

#### 2.1.2 1,4-Addition

Ub-VS bears a vinyl methyl sulfone group at the C-terminus and can react with the active Cys to form a stable 1,4-adduct.^15,22^ Other probes that use the 1,4-addition mechanism include Ub-VME, Ub-VCN, Ub-Dha, Ub-OEtVS, Ub-VSPh, and Ub-AVSN.^22–31^ One useful feature of the 1,4-addition probes is that, unlike Ub-Prg, the thiol-reactive group can be readily incorporated at the site between two Ubs, facilitating the capture of DUBs that specifically hydrolyse different Ubs.

#### 2.1.3 Nucleophilic substitution

Ub-based chemical probes that react with the active Cys through nucleophilic substitution include Ub-Cl, Ub-Br2, Ub-Br3, and Ub-AOMK.^22,27,32^ In addition to capturing DUBs of USP and UCH families, this category of probes is also useful for identifying the ovarian tumour proteases (OTU) family DUBs.^22^

### 2.2 Probes capturing Ub interactors

The recruitment and binding of different Ubs by the Ub interactors is usually non-covalent in nature. The development of Ub-based chemical probes to capture or enrich Ub interactors can inform the study of these recognition events.

#### 2.2.1 Pull-down probes

**Fig. 4:**
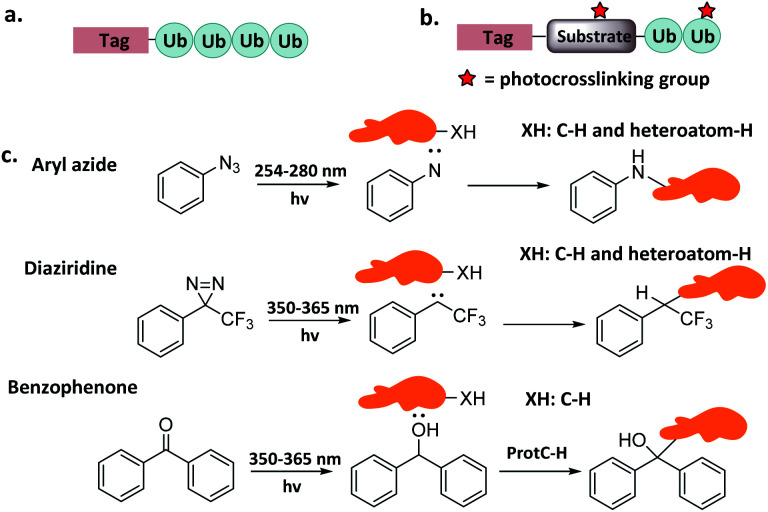
Probes capturing Ub interactors. (a) Pull-down probes. (b) Photo-crosslinking probes. (c) Photocrosslinking groups and related reactions.

Ub interactors containing one or more Ub binding domains (UBD) can bind to Ubs through non-covalent interactions. This is the basis of a simple but effective strategy, wherein Ub pull-down probes are used to capture or enrich Ub interactors from the cell lysates. Such probes usually contain one or more Ub units and an enrichment/purification tag.^33–40^ Ub units can be either natural or mimetics that do not impact the interaction with linkage-specific interactors (Fig. 4a).

#### 2.2.2 Photocrosslinking probes

For the Ub interactors that are difficult to capture using pull-down probes (because of weak interactions), a photocrosslinking group can be installed on the Ub chain (Fig. 4b). UV light is used to induce a covalent bond between the probe and the interactor to capture the latter.^41–43^ Common photoactive groups include aryl azide, diaziridine, and benzophenone. The aryl azide group can generate a nitrene intermediate, though may be reduced under the physiological conditions, resulting in low crosslinking efficiency.^44^ In contrast, diaziridine can generate a more active carbene intermediate able to insert into various C–H bonds with high labelling efficacy. Finally, the wavelength to activate benzophenone is relatively long (350–365 nm), which is less damaging to protein^44^ (Fig. 4c).

## 3. Synthesis of Ub-based chemical probes

Synthesis of the Ub-based chemical probes usually involves two steps: first, synthesis of Ub conjugate module; second, incorporation of the active group. Ub conjugates are usually difficult to obtain through direct recombinant expression, and therefore need to be synthesized through chemical means.

### 3.1 Total chemical synthesis of Ub conjugate module

**Fig. 5:**
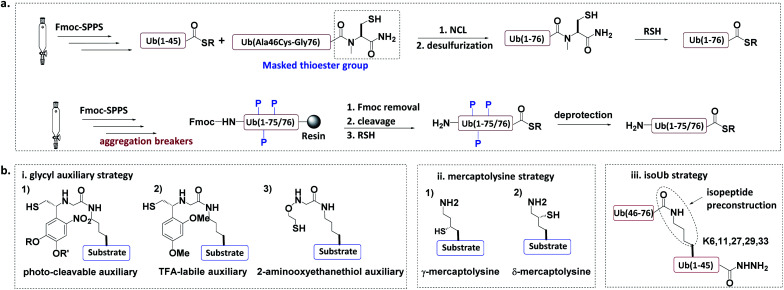
Chemical synthesis of Ub conjugate. (a) Total chemical synthesis of Ub conjugate. (b) Construction of isopeptide bonds.

In the total chemical synthesis of Ub conjugates, the target Ub conjugate is usually divided into separate segments at a site close to the isopeptide. Ub conjugates are generally synthesized from a donor Ub thioester and a substrate protein bearing a “Cys-like” auxiliary group at or close to the isopeptide Lys. After their ligation, the “Cys-like” auxiliary is removed from the ligated product to yield the target Ub conjugate^45^ (Fig. 5).

#### 3.1.1 Total chemical synthesis of Ub donor thioesters

Fmoc solid-phase peptide synthesis (SPPS) can be used to synthesize peptide thioesters, but this method is usually limited to peptides of up to 50 amino acids^46–50^ (Fig. 5a). Since Ub contains 76 amino acids, its direct synthesis through Fmoc-SPPS is difficult. To overcome this problem, one approach is to divide the Ub into two segments which can be separately synthesized, and then ligated together. As an example, Brik *et al.* used Fmoc SPPS to synthesize two segments, *i.e.* Ub(1-45)-thioester and Ub(A46C-G76) with a masked thioester. Native chemical ligation (NCL) between the two segments led to the formation of full-length Ub. After the conversion of Cys46 to Ala through desulfurization and activation of the masked thioester, the target Ub-thioester was successfully obtained. In addition, a Ub-hydrazide can be synthesized in a similar two-segment strategy, and used as a thioester equivalent.^51–61^

Another approach for the synthesis of long peptides on the solid support is to incorporate “aggregation breakers” such as pseudoproline and dimethoxybenzyl dipeptides,^62^ both of which disrupt the aggregation of the nascent peptide on the resin surface. Using this strategy, Ovaa *et al.* successfully synthesized Ub thioesters and even SUMO2 (92 amino acids).^63^

#### 3.1.2 Construction of isopeptide bonds

Several methods have been developed to introduce the “Cys-like” auxiliary group at or close to a specific Lys residue in the substrate protein (Fig. 5b).

##### 3.1.2.1 Glycyl auxiliary strategy

A photo-cleavable thiol-containing glycyl auxiliary can be introduced to the Lys of the substrate protein through SPPS.^64^ The auxiliary reacts with the Ub-thioester through NCL, and then the auxiliary can be selectively removed *via* photolysis to generate the desired ubiquitinated substrate. Two alternative auxiliaries are 1-(2,4-dimethoxyphenyl)-2-mercaptoethyl, which can be removed by trifluoroacetic acid after ligation;^63,65^ and a 2-aminooxy-ethanethiol auxiliary, which exhibits faster ligation.^66^

##### 3.1.2.2 Mercaptolysine strategy

Brik and Liu groups designed δ- and γ-mercaptolysine and incorporated them into substrate proteins.^67,68^ Both δ- and γ-mercaptolysine can be ligated with a Ub-thioester, leading to the formation of an isopeptide bond after desulfurization. Compared to the glycyl-auxiliary strategy, the mercaptolysine uses a primary amino group to participate in the ligation and has a faster ligation speed.

##### 3.1.2.3 IsoUb strategy.^69^

This strategy employs the 76-residue isoUb unit, which is made from two adjacent Ub segments, each of which contains an N-terminal Cys and a C-terminal hydrazide to facilitate ligation. Multiple isoUb units can be assembled through sequential hydrazide-based NCL. Unlike native Ub, the isoUb unit does not aggregate, and can therefore be readily synthesized. Furthermore, the ligation of isoUb takes place at Cys with less hydrolysis by-products. Using the isoUb strategy, a K11/K48-branched hexa-Ub (456 amino acids) was synthesized.

### 3.2 Protein semi-synthesis of Ub conjugate module

The total chemical synthesis may present technical challenges to some biochemistry-oriented laboratories. Protein semi-synthesis methods may be a useful alternate.^70–74^

#### 3.2.1 Recombinant production of Ub thioesters

**Fig. 6:**
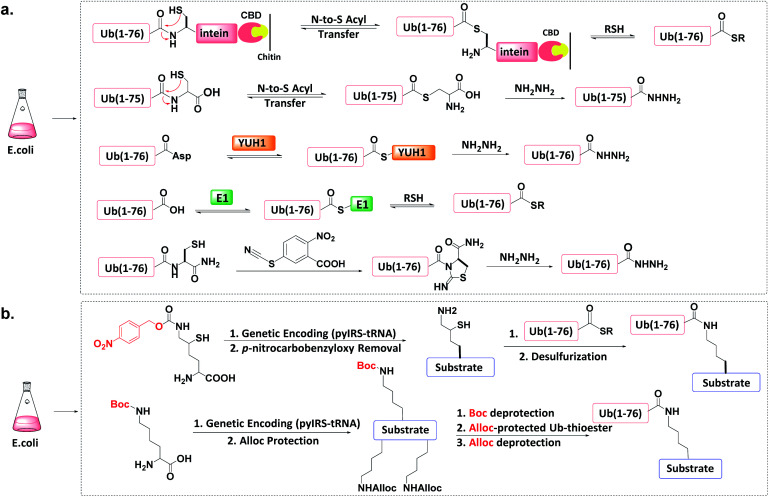
Protein semi-synthesis of Ub conjugate. (a) Recombinant production of Ub thioesters. (b) Recombinant introduction of isopeptide bonds.

Ub thioesters can be produced through intein splicing, wherein an expressed protein undergoes an intramolecular *N*-to-*S* acyl transfer to produce a protein thioester with the expulsion of the intein^75,76^ (Fig. 6a). To obtain a Ub thioester, Ub needs to be fused to the N-terminus of the intein (where C-terminal Asn is mutated to Ala), and the chitin binding-domain (CBD) is fused to the intein C-terminus. Upon the addition of thiol (to promote the rearrangement), the intein incorporating the Asn-to-Ala mutation cannot complete the entire splicing process. Instead, the intermediate would undergo *trans*-thioesterification with the external thiol, affording Ub-thioester as a released product (usually 4–5 mg L^−1^ of LB medium).

Other methods can produce recombinant Ub-hydrazide (as a Ub thioester equivalent). First, Macmillan *et al.* discovered that peptidyl C-terminal Cys can undergo *N*-to-*S* acyl transfer to generate a transient thioester.^77^ After adding hydrazine to Ub(1-76C), *N*-to-*S* acyl transfer generates Ub(1-75)-hydrazide with a yield of *ca.* 50 mg L^−1^ of LB medium. Second, Liu *et al.* discovered that the Ub hydrolase YUH1 can hydrolyse Ub analogues to form thioester intermediates.^78^ By appending Asp to the Ub C-terminal and treating Ub(1-77D) with YUH1 and hydrazine, Ub(1-76)-hydrazide was obtained in a high yield (*ca.* 30–40 mg L^−1^ of LB medium). Third, the transesterification of E1 and Ub to form an active E1-Ub thioester can be intercepted by the addition of thiol, leading to the formation of Ub (1-76)-thioester in good yields (*e.g.*, 50 mg L^−1^).^36,79,80^ Finally, Liu *et al.* found that a small molecule cyanylating reagent (2-nitro-5-thiocyanatobenzoic acid) can modify a recombinant protein at its C-terminus *via* nucleophilic acyl substitution, generating a protein hydrazide if the nucleophile is hydrazine.^81^

#### 3.2.2 Recombinant introduction of isopeptide bonds

Substrate proteins containing “Cys-like” auxiliary groups can also be obtained through *E. coli* expression (Fig. 6b). Chin *et al.* used a specific pyrrolysyl-tRNA synthetase and tRNA pair to incorporate photo-caged δ-mercaptolysine into the recombinant proteins.^82^ After deprotection by light, a substrate protein containing a “Cys-like” auxiliary was obtained and used for the construction of isopeptide bonds.

An unnatural amino acid strategy has also been used to introduce a side-chain Boc-protected Lys into the substrate protein.^83^ The remaining free amines on the substrate protein and on the donor Ub-thioester are chemically masked by another protecting group such as allyloxycarbonyl (Alloc). After Boc deprotection, Ub-thioester was reacted with the substrate protein forming an isopeptide bond through Ag-catalysed thioester-amine condensation. Finally, the removal of Alloc led to the formation of the target Ub conjugate.

### 3.3 Biorthogonal synthesis of mimic Ub conjugates

**Fig. 7:**
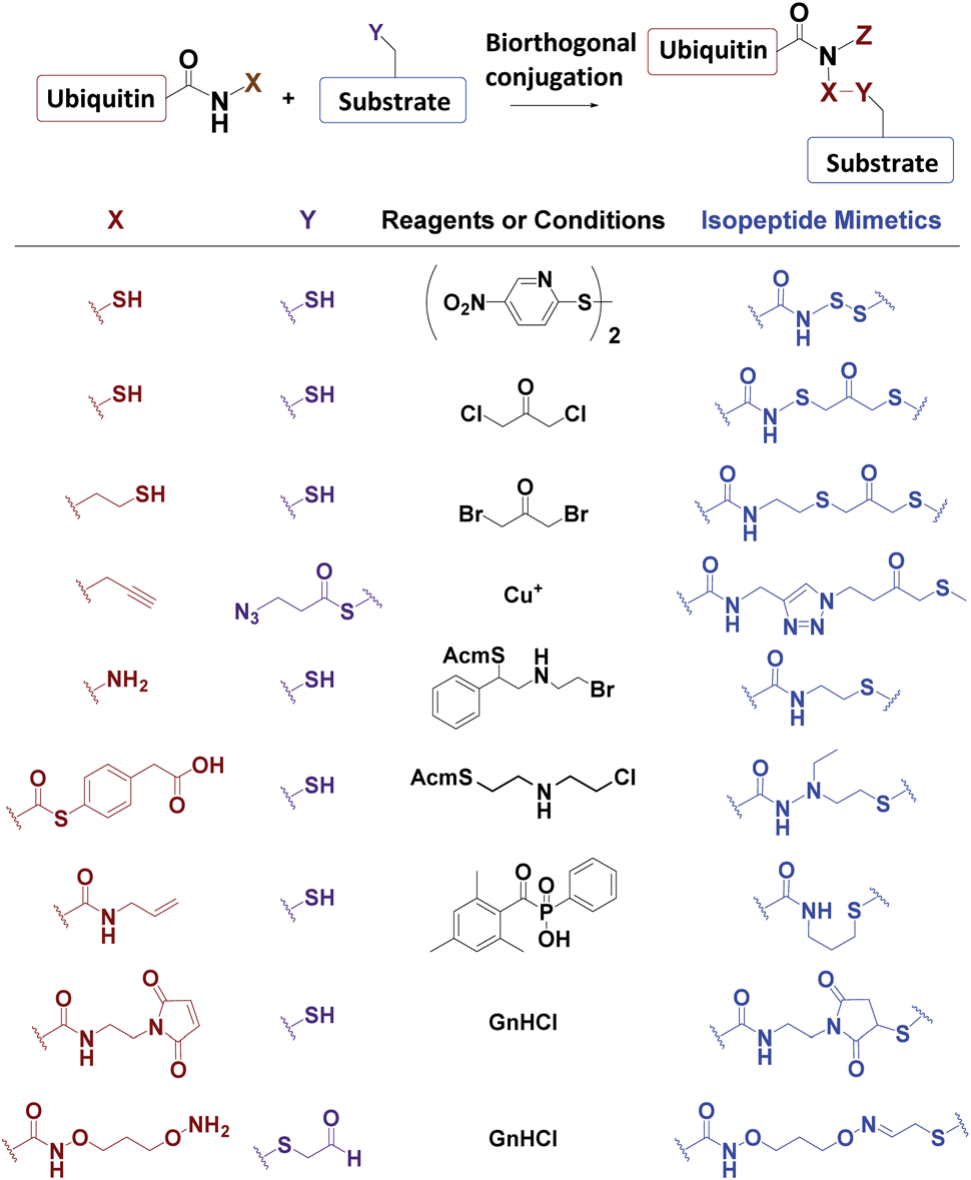
Biorthogonal synthesis of Ub mimetics.

Ub conjugates incorporating isopeptide bond mimetics can also be synthesized from the expressed Ub^70–73,84,85^ (Fig. 7).

#### 3.3.1 Disulfide crosslinking

2-Mercaptoethyl amide can be introduced to the C-terminus of Ub to obtain Ub-SH. Next, 5,5′-dithiobis(2-nitrobenzoic acid) was added to Ub-SH generating a high-energy disulfide. This intermediate was then reacted with the substrate protein bearing a Lys-to-Cys mutation at the desired position, generating a Ub-substrate disulfide adduct.^86,87^ The protein precursors used in this strategy can be readily obtained *via* expression, and the coupling step is usually efficient. One disadvantage is that the S–S isopeptide mimetics may be unstable under reducing conditions.

#### 3.3.2 1,3-Dichloroacetone crosslinking

The Lys (to be ubiquitinated) on the substrate protein is mutated to Cys, and the Gly76 of the donor Ub is mutated to Cys. When 1,3-dichloroacetone is added, the substrate forms a crosslinked adduct with Ub.^88,89^ The advantage of the strategy is that the protein precursors are easy to express, and the isopeptide mimetics are stable in reducing conditions. Nonetheless, the crosslinking process may produce two self-crosslinking by-products. Matthew *et al.* proposed the use of 1,3-dibromoacetone in a stepwise crosslinking, which generates a single crosslinked product with a high yield.^90^

#### 3.3.3 Azide–alkyne cycloaddition

Cu-catalysed azide–alkyne cycloaddition (CuAAC) reactions can be used to construct isopeptide bond mimics.^38,39,91^ Introduction of the azide into the Ub C-terminal was accomplished through unnatural amino acid insertion, and then the alkyne was introduced into the substrate Lys through bio-orthogonal reactions. The Ub and substrate were ligated in the form of triazole *via* CuAAC reaction. The reaction has good substrate adaptability, and the triazole isopeptide bond mimetic is resistant to DUB hydrolysis.

#### 3.3.4 Aminoethylation-NCL coupling

To obtain a Ub conjugate incorporating a closer mimic of the native isopeptide bond, a bifunctional handle was appended onto a substrate protein through aminoethylation reaction with Cys.^36,78^ Ligation of the substrate with the Ub-thioester using NCL followed by auxiliary removal led to the formation of the isopeptide bond mimetics. Two different bifunctional handles were developed: one that results in mimetics bearing only one atom different from the native isopeptide bonds, which may provide ideal tools for biochemical and structural studies;^78^ and the other producing an *N*-ethyl isopeptide bond that is stable to DUB and therefore, useful for screening Ub chain binding proteins from cell lysates.^36^

#### 3.3.5 Thiol-ene coupling strategy

Thiol-ene coupling can be used for nonenzymatic synthesis of Ub chains. This strategy requires the use of a Ub variant bearing a C-terminal allyl amine appendage, which can act as E2-*S*-Ub intermediate. Cys is used as the lysine surrogate providing linkage specificity. Free-radical thiol-ene polymerization will lead to the formation of non-native isopeptide bonds between Ubs.^92^ Thiol-ene coupling strategy can be applied to the synthesis of site-specific isopeptide, Ub oligomers, and also branched Ub trimers.^93^

#### 3.3.6 Maleimide coupling

Brik *et al.* developed a coupling strategy based on maleimide. First, Ub(1-75)-NHNH_2_ is converted to Ub(1-75)-maleimide with the addition of NaNO_2_ and *N*-(aminoethyl) maleimide. Then, the olefin group on the maleimide can then be linked to another protein.^94^

#### 3.3.7 Oxime ligation

Brik *et al.* also reported a strategy based on a set of well-defined conjugates bearing an oxime bond. Ub-methyl 3-mercaptopropionate thioester is incubated with 1,2-bisaminoxy ethane and the Ub C-terminus is modified with an oxyimino group. Through isopeptide ligation *via* an oxime bond, polyUb chains can be synthesized.^95^

### 3.4 Incorporation of chemically reactive groups

Ub-based chemical probes incorporate two types of active groups: one to capture the active Cys residue, and the other to capture protein-interacting interface amino acids.

#### 3.4.1 Incorporation of chemical groups to capture cysteine

**Fig. 8:**
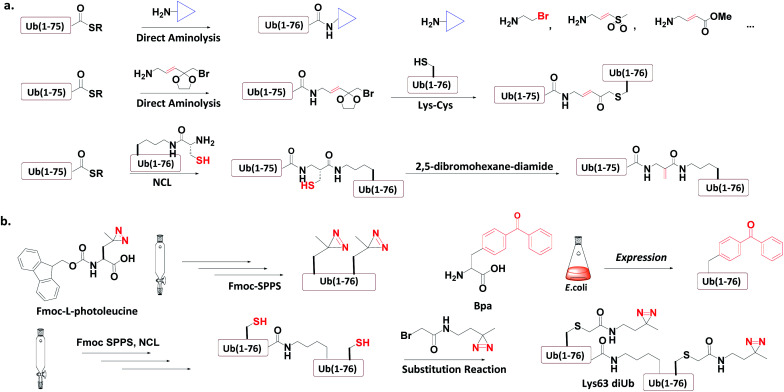
Incorporation of chemically reactive groups. (a) Strategies to capture the Cys residue. (b) Strategies to incorporate photocrosslinking groups.

Three strategies have been used to incorporate the chemical groups to capture the Cys residue (Fig. 8a).

##### 3.4.1.1 Direct aminolysis

Kessler *et al.* used the protein semi-synthesis method to prepare an HA-labelled Ub-thioester and then introduced the active group to the C-terminus of Ub through direct aminolysis.^22^ This approach was used to make a series of monoUb active probes including Ub-Michael acceptor and Ub-halides. Zhuang *et al.* attached a bifunctional linker containing a masked Michael acceptor at the C-terminus of the remote Ub thioester.^96^ The ketal group in the linker was then deprotected to generate an α-bromo ketone. After a thiol substitution reaction was carried out with the α-bromo ketone intermediate, a diUb probe was obtained.

##### 3.4.1.2 NCL

Brik *et al.* mutated the remote Ub's Gly76 to Cys and loaded it onto the Lys side chain amino group of the receptor Ub through SPPS.^24^ After ligation with the remote Ub thioester through NCL, 2,5-dibromohexanediamide was used to convert the Cys76 residue to Dha. In this manner, a diUb probe was obtained. Ovaa *et al.* designed an NCL ligation handle, which allows the ligation of Ub onto a protein. This building block was loaded on the proximal Ub and then reacted with Ub(1-75)-thioester to construct *via* NCL. Through *in situ* selective thiol elimination, a diUb probe was constructed.

#### 3.4.2 Incorporation of photo-crosslinking groups

Three methods have been used to introduce the photocrosslinking groups into the Ub conjugates (Fig. 8b).

##### 3.4.2.1 Direct SPPS

Ovaa *et al.* introduced photoleucine containing a photocrosslinking group into the sequence of Ub through SPPS, and then enzymatically assembled it into a Ub chain to generate a photocrosslinking polyUb probe.^97^

##### 3.4.2.2 Insertion of unnatural amino acids

Virdee *et al.* used an evolved engineered pair of tRNA and aminoacyl-tRNA synthetase to embed *p*-benzoyl-l-phenylalanine (Bpa) into the sequence of Ub.^41^ This Ub mutant was covalently put onto the active Cys residue-to-Lys mutated E2 to construct an E2-Ub photocrosslinking probe.

##### 3.4.3.3 Post-ligation thiol modification

Tian *et al.* mutated Ub's Ile44 to Cys and obtained a diUb conjugate through hydrazide based NCL.^98^ The two Cys residues of the synthetic conjugate were then modified with photocrosslinking groups (diazirine or aryl azide) through selective substitution reaction, leading to the generation of a diUb photocrosslinking probe.

## 4. Applications of Ub-based chemical probes

### 4.1 MonoUb/UbL probes

#### 4.1.1 Identification of DUBs

As early as 1987, Ubal was shown to be an effective probe for capturing DUBs.^99^ In 1997, Cohen *et al.* captured a 37 KD subunit with previously unknown DUB activity from proteasome 19S regulatory complex using an I^125^-isotope labelling Ub-CN probe.^99^ Subsequently, it was identified as UBCH37.^100,101^

The covalent product of Ub-CN and DUB was not compatible with the reducing conditions of SDS/PAGE. In 2001, Ploegh *et al.* developed Ub-VS that can form irreversible adducts.^15^ This probe reacted efficiently with UCH-L3, and also showed high activity with an array of Ub carboxyl terminal hydrolases such as Ubp1, Ubp2, Ubp6, Ubp15, and Yuh1. Using this probe, a unique DUB, *i.e.* USP14, was identified in the mammalian 26S proteasome. In 2002, more monoUb probes were developed, including HA-Ub-Cl, HA-Ub-Br2, HA-Ub-Br3, HA-Ub-VS, HA-Ub-VME, HA-Ub-VSPh, and HA-Ub-VCN.^22^ These probes exhibited distinct DUB profiling, enabling the identification of DUBs by affinity-tag based mass proteomics (AP-MS). A total of 23 active DUBs were identified in EL4 cells. HSPC263, a protein labelled by HA-Ub-Br2, had no sequence homology with known DUB and was determined to belong to a new OUT family of DUB.

To overcome false positives caused by non-covalent interactions and non-selective capturing, Wertz *et al.* developed a ‘reactive-site-centric chemoproteomics’ method for the detection of probe-labelled residues by enhanced monoUb-probes. A C-terminal alkyne was added and attached with a cleavable biotin-azide tag *via* click reaction.^102^ Using the new probe, a previously unannotated DUB, ZUFSP with high Lys63-linked specificity was identified.^103^

Finally, mono-Ub probes can be used for the identification of DUBs in defined cellular compartments.^104^ For instance, HA-Ub-VS was used to study the function of USP7 during lipogenesis. Transcriptional coregulator Tip60, whose expression is regulated by polyubiquitination on multiple sites, plays a key role in adipocyte differentiation. Combining activity monitoring using Ub-VS and further *in vitro* and *in vivo* experiments, it was found that early adipogenesis is regulated through USP7-mediated de-ubiquitination of the Tip60.^105^

#### 4.1.2 Screening of inhibitors for DUBs

Mono-Ub probes can be used for screening of small-molecule inhibitors of DUB. For example, the inhibition of USP7 enables activation of the tumour suppressor p53 – a typical ‘undruggable’ target in various cancer cells. In the effort to find and optimize potent, specific inhibitors of USP7, several probes such as Ub-VS, Ub-Br2, and Ub-PA were used to measure their potency by the activity competitive assay.^106–108^ Additionally, fluorescently labelled mono-Ub probes for visualizing activity of Ub-related enzymatic can be used to validate drug targets and design novel inhibitors. Ub aminomethyl coumarin (Ub-Amc) was the first fluorescent probe to report DUB activity.^109^ Ub-Rho110, bearing a substituted fluorescent molecule rhodamine-110, was also developed for high-throughput screening.^110^

#### 4.1.3 Elucidation of the enzymatic mechanisms

In 2005, Gaudet *et al.* solved the crystal structure of UCH-L3 and Ub-VME complex (1.45 Å) and found that a loop region (residue 147–167) of complexed UCHL3 exhibited a significant conformational rearrangement.^17^ Comparison with UCHL3 apo state showed that the loop is invisible and may traverse the active site cleft; but once bound it changed to a well-ordered structure, exposing the interior active site. This has been proposed as the key to Ub binding in the whole family members of UCH.^17^ Further studies showed that changes of the length and flexibility of the active-site crossover loop also contribute to the substrate specificity of UCH family.^111^

Another study elucidated the extent to which activation and inhibition of UCH-L5 was tuned by its two adaptor RPN13 and INO80. Both adaptors bind to UCH-L5 as demonstrated by the crystal structures.^18^ However, the consequences of binding are different: RNP13 exerts activation activity, whereas INO80 inhibits activity. To explain regulatory mechanisms, Ub-Prg was used to capture the catalytic conformation by which RPN13^DEUBAD^ activates UCH-L5; and the catalytic conformation that truncated INO80^DEUBAD^ lacks to inhibit UCH-L5. On the basis of multiple intermediate structures, it was found that RPN13^DEUBAD^ activates UCH-L5 by positioning its domains while INO80^DEUBAD^ inhibits UCH-L5 by blocking Ub binding.^18^

Finally, monoUb probes can also be employed to study the mechanism for activation and thioester bond formation in E1s. Two mono-Ub probes were used to capture E1 intermediates,^31^ including Ub-AMSN probe to mimic the adenylate intermediate, and Ub-AVSN to mimic the tetrahedral intermediate. These two structures have shown significant conformational changes, from an open conformation before release of pyrophosphate to a closed conformation required for thioester bond formation.^19^ Similar probes have also been applied to capture Sumo E1's active intermediates and study structural mechanism.^19^

#### 4.1.4 Capturing of UbL hydrolyses/interactors

**Fig. 9:**
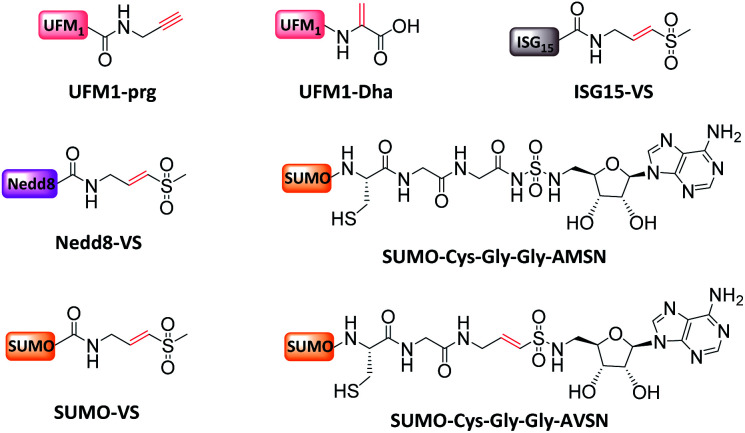
MonoUbL probes used to study UbL enzymes and interactors.

Ovaa *et al.* introduced a vinyl sulfone moiety to the C-terminus of Nedd8, ISG15, and SUMO-1, and proved that these UbL-VS probes enable the exploration of the Ubl enzymatic system in complex cellular environments^16^ (Fig. 9). Subsequently, targeting another Ubl, ubiquitin-fold modifier 1 (UFM1), Ovaa *et al.* developed another two active probes based on the reactive groups of Dha and PA, and verified their activity and specificity *in vitro* and in cell lysate^112^ (Fig. 9).

Mootz *et al.* prepared a SUMO-1 photo-crosslinking probe by introducing Bpa at the Arg50 of Sumo-1.^113^ The active site-independent probe captured the known effector protein RanBP2 in complex cell lysate, portending its use for profiling the sumo-related pathways of real cell systems.

### 4.2 Ub/UbL chain probes

#### 4.2.1 Profiling DUB's Ub chain linkage selectivity

**Fig. 10:**
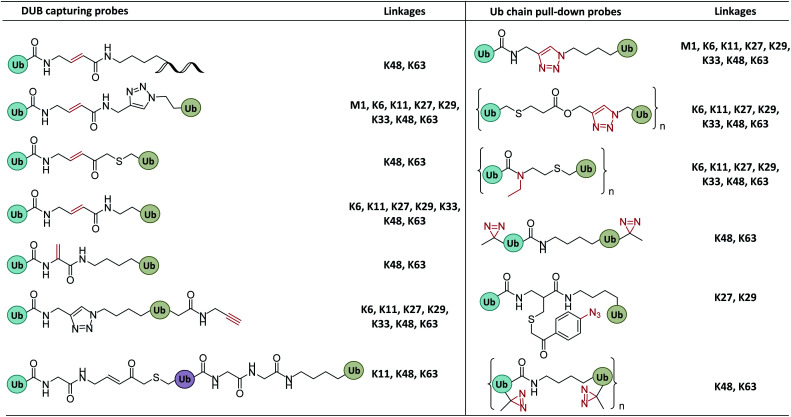
Structures of Ub/UbL chain probes used to identify chain-specific DUBs, study the mechanism by which Ub chains are selectively bound and cleaved by DUBs, profile Ub chain interactors, and monitor changes of Ub chain conformation in recognition.

The earliest attempt at profiling DUB's Ub linkage selectivity was carried out by Franke *et al.*, who used a Ub-peptide to mimick K48- and K63-diUbs^33^ (Fig. 10). Pull-down tests in cell lysates indicated that USP5, USP7, UCH-L3, and UCH-L5 showed a preference for K48 Ubs, whereas USP19 and USP38 showed a preference for K63 Ubs. Later, Kessler *et al.* developed full-length diUb probes with a triazole to mimic isopeptide bonds.^114^ Global profiling and proteome analysis in cell lysates showed a total 29 DUBs trapped by these probes, including 18 USPs, 4 Ub C-terminal hydrolases (UCHs), 5 OTUs, and 1 Machado–Josephin domain (MJD). MS-based quantitation showed that most USPs have little selectivity towards diUbs while OTU family DUBs exhibited highly chain selectivity, *e.g.* OTU7B shows a strong preference for K11 diUb probes and OTUB1 favoured K48 diUb.

To circumvent the steric hindrance of the diUb probes, other diUb probes incorporating a Michael acceptor that more closely resemble native diUbs were developed. For example, Ovaa *et al.* used K11 and K48 diUb probes to label Cezanne (a K11-specific DUB that regulates cellular inflammation, NF-κB signalling and T cell activation) and found that Cezanne can be exclusively labelled by K11 diUb active probes.^62^ Brik *et al.* found that IsoT, USP2, OTUB1, and OTUB2 hydrolyse K48 diUb based on dehyrdoalanine (Dha) probe.^24^

Nonetheless, activity-based diUb probes cannot readily capture K27-active DUBs due to the steric hindrance of the K27 isopeptide bond. Li *et al.* reported a photocrosslinking K27 diUb probe bearing an aryl azide group,^115^ which probe captured K27-selective DUBs from cell lysates (OTUD2 and USP13). A similar photocrosslinking K29-diUb probe also captured K29 specific DUBs (ZRANB1 and OTUD2).

#### 4.2.2 Mechanism for Ub chains binding and cleavage

Komander *et al.* used Lys11 diUb probes to illustrate a non-canonical *k*_cat_-driven catalytic cycle of Cezanne. A K11 diUb probe was used to react with Cezanne to form a Cezanne-K11 diUb complex for crystallization. By comparing the structures of Cezanne alone (2.2 Å) and in complex with K11 diUb probe (2.8 Å),^34^ as well as the results of dynamic H/D exchange mass spectrometry experiments, a three-step hydrolytic cycle was postulated accounting for Cezanne's Lys11 specificity. First, the priming of distal Ub at the S1 site of Cezanne opens the autoinhibition of apo-state by releasing Cys-loop. Next, a transient conformation is remodelled to form S1′ site *in situ* only if Lys11-linked diUb is bound, relying on the key interaction Lys33 (in proximal Ub) and Glu157 (in Cezanne). Finally, upon hydrolysis, the S1′ site is destroyed the proximal Ub expelled, leading to a rearrangement back to the apo-Cezanne.

Severe acute respiratory syndrome coronavirus papain-like protease (SARS PLpro) is a DUB which recognizes K48 polyUb chains *via* at least two binding sites of S2–S1, rather than S1–S1′.^116^ To elucidate the structural basis of this selectivity, a distal diUb^K48^ probe was developed to crosslink SARS PLpro, and the covalent complex crystalized and solved at 2.85 Å.^117,118^ It was found that at least three Ub-binding sites cooperated to cleave K48 Ub. The S1 and S2 sites of PLpro remodelled K48 diUb to an extended conformation, resulting in K48 specificity.

Finally, a triUb probe with well-defined cross-linking site can be used to investigate whether a DUB performs endo- and exo-Ub chain cleavage.^35^ For example, USP9X, a DUB regulating multiple important cellular processes including apoptosis and stem cell self-renewal, displays multiple Ub-binding sites and selectivity for K11, K48, and K63 Ubs. In order to investigate its cleavage mode, Zhuang *et al.* developed K11, K48, and K63 tri-Ub probes, in which the active group is located at the isopeptide bond between the distal and middle Ubs. Experiments with these probes revealed that USP9X cleaved the K11, K48, and K63 Ubs in different modes (*i.e.* endo, exo, and mixed).

#### 4.2.3 Profiling Ub chain interactors

DiUb probes bearing a triazole structure were used by Ovaa *et al.* to identify Ub linkage interactors from cell lysates.^37^ The triazole bond is resistant to endogenous DUBs. Eight different diUb probes were examined to obtain a global Ub interactome by Ub interactor affinity enrichment-mass spectrometry (UbIA-MS). TAB2 ZNF and TAB3 ZNF domains were identified as new interactors of K6 diUb, while UCHL3 (Ub carboxyl-terminal hydrolase isozyme L3) was found to be a new interactor for K27 Ubs. By comparing the differences of Ub interactors in HeLa cells, mouse embryonic stem cells (ESCs), and neuronal precursor cells (NPCs), the authors found that K27, K29, K33, and K6 Ub interactors showed cell-type dependence.

In another study of Ub interactors, Stengel *et al.* developed polyUb probes bearing up to ten Ub units.^38^ These probes were applied to proteomic detection, from which 70, 44, and 37 proteins were found to be interactors of K27, K29, and K33 Ub, respectively. The authors also used gel eluted liquid fraction entrapment electrophoresis to separate the Ubs into Ub_2,_ Ub_4_, Ub_6+_ to examine their interactors separately.^39^ Significant differences were observed in the interactors of Ubs with different lengths. For example, Ub-associated domain-containing protein 1 (Ubac1), RING finger 123 (RNF123), and USP15 only interact with long Ub chains (Ub_4_, Ub_6+_).

In addition, triazole-free DUB-resistant Ub probes were developed containing *N*-ethyl isopeptide bonds.^36^ These probes were used to profile the interactome of K29 Ubs, which captured some new interactors not identified by the triazole probes. The main limitation of the Ub pull-down probes is that weak interactors cannot be captured. Tian *et al.* reported diazirine-based photoaffinity probes that can capture K48- and K63-Ub interactors in cell lysates.^98^ Meanwhile, Glickman *et al.* developed photo-crosslinking polyUb probes for detecting proteasome subunits that interact with Ubs.^97^

#### 4.2.4 Monitoring changes of Ub chain conformation

Komander *et al.* designed M1, K63 and K48 diUb probes containing a FRET dye pair.^119^ These probes were used to measure the conformational change in the presence of recognition protein. It was found that Ub chains are present in a multi-conformational equilibrium in solution. K63 and M1 diUb exhibited an extended “open” and “semi-compact” conformation, while K48 diUb barely exhibited any “open” conformation in isolation. Some DUB such as AMSH-LP (for K63 diUb), OTUB1 (for K48 diUb) and USP21 (for M1 and K63 diUb) chose the pre-existing conformation to achieve the cleavage, but USP21 must remodel K48 diUb into an “open” conformation in order to undertake enzymatic activity.

### 4.3 Ub/UbL substrate probes

#### 4.3.1 Capture and study DUBs

**Fig. 11:**
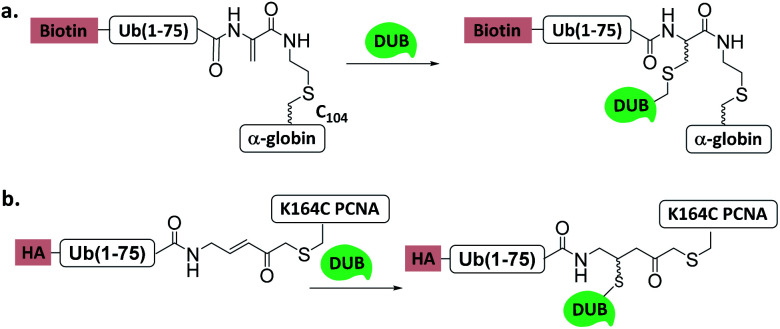
Ub/UbL substrate probes used to capture and/or study DUBs. (a) α-globin-Ub-Dha. (b) K164C Ub-PCNA-MAL.

Brik *et al.* reported a α-globin-Ub-Dha probe for the identification of α-globin specific DUBs^30^ (Fig. 11a). The probe was incubated with haemoglobin-depleted erythrocyte lysate, and after affinity purification, 29 proteins were found, including 4 DUBs (USP15, USP14, USP5, and UCHL3). Subsequent experiments confirmed USP15 as a DUB of α-globin. Brik *et al.* also reported the chemical synthesis of an H2A-Ub-Dha probe, and successfully assembled the probe into the nucleosome. Biochemical studies showed that the H2AK119Ub specific DUB Calypso/ASX can be efficiently captured by this probe.^25^

Another Ub-substrate probe, Ub-PCNA-MAL, was developed by Zhuang *et al.*^40^ (Fig. 11b). Two ubiquitination sites on yeast PCNA (K107 and K164) were examined for the probes. Pull-down experiments in yeast lysate and quantitative mass spectrometry analysis showed that both probes could capture a variety of DUBs. Ubp3 and Ubp10 were found to be specific for K164. This finding indicates that DUBs can distinguish ubiquitination sites on a substrate protein.

#### 4.3.2 Capture and study E3s

**Fig. 12:**
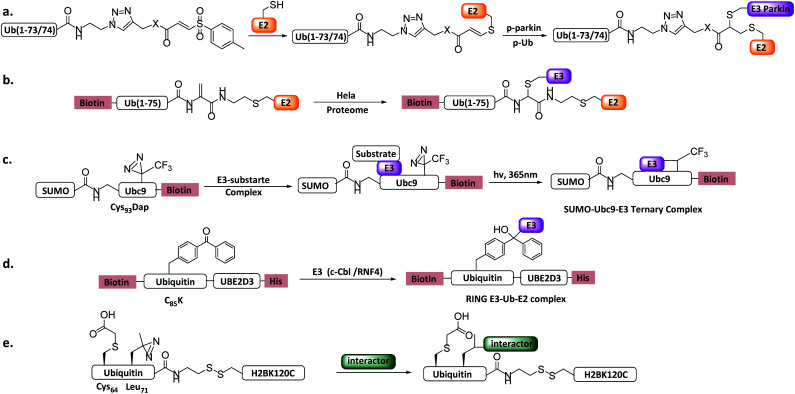
Ub/UbL substrate probes. (a) E2-Ub-AVS. (b) E2-Ub-Dha. (c) E2-SUMO. (d) E2-Ub photocrosslinking probe. (e) Ub-nucleosome probe.

Virdee *et al.* developed an activity-based probe E2-Ub-AVS for RBR E3s (Fig. 12a).^28^ The probe could react with the RBR E3 parkin in a cysteine dependent manner. *In vitro* experiments showed that Ser65-phosphorylated parkin (p-parkin) reacted with the probes only in the presence of Ser65-phosphorylated ubiquitin (p-Ub), indicating that p-Ub is a prerequisite for p-parkin sustaining its transthiolation activity. A panel of parkin mutations associated with Parkinson's disease was profiled with the probes and revealed that nearly all contributed to defects in transthiolation activity. The probes were further applied to cell extracts or patient tissues to investigate parkin activation. Licchesi *et al.* later showed these probes react with HECT E3, such as NEDD4, UBE3C, and HECTD1.^120^

More recently, a variant of the probe E2-Ub-AVS was developed, which showed improved labelling efficiency and was applied to profiling HECT or RBR E3s in neuroblastoma SH-SY5Y cell extracts. About 40 HECT/RBR E3s were profiled, consisting of 80% known HECT/RBR ligase. Notably, 33 RING ligases were also enriched, of which a novel RING ligase MYCBP2 was discovered to utilizes a unique RING-Cys relay (RCR) mechanism mediating the transfer of Ub onto the substrate threonine and serine residues. *In vitro* and *in vivo* experiments verified NMNAT2 (nicotinamide mononucleotide adenyltransferase) was the substrate of MYCBP2.^29^

Shi *et al.* reported an E2-Ub-Dha probe (Fig. 12b), containing UBE2D2, an Ub moiety and a Dha reacting group.^121^*In vitro* labelling assay showed that the probe can react with HECT E3 NEDD4 and UBE3C efficiently. *In vivo* profiling using HeLa cells identified several HECT E3s including NEDD4, UBE3C and HUWE1. Moreover, two RBR E3 (ARIH1, ARIH2) and several RING E3s were also enriched, similar to E2-Ub-AVS probe.

Other Ub substrate probes have been designed to capture RING-type E3s. Unlike HECT/RBR E3s that can be capture by activity-based probes, RING-type E3s do not bear a catalytic Cys. Bode *et al.* developed a photo-reactive E2-SUMO probe to trap RING type SUMO E3 ligase.^42^ C93 of SUMO E2 Ubc9 was mutated to 2,3-diaminopropionic acid to form a stable amide-linked E2-SUMO conjugate (Fig. 12c), and diazirine was introduced to Ubc9 F22 to trap E3 ligases. When the probe was incubated with cell lysate, SUMO E3 ligase RanBP2 can be enriched. Moreover, Virdee *et al.* developed an E2-Ub photo-reactive probe (Fig. 12d), in which Bpa was genetically introduced. The activity of the probe was demonstrated by crosslinking with RNF4 in a SUMO chain dependent manner.

#### 4.3.3 Study histone PTM crosstalk

Dot1L-mediated methylation of histone 3 K79 was reported to be stimulated by ubiquitination of histone 2B at K120.^122,123^ To decipher the activation mechanism, Muir *et al.* developed a photo-reactive Ub-nucleosome probe (Fig. 12e).^43^ The probe was incubated with Dot1L, and after UV irradiation, the crosslinked species were analysed by LC-MS/MS. The N terminus of H2A was found to interact with Ub. A foot-printing assay using an Ub-nucleosome probe containing H2B-Ub and H3K79C (diazirine) revealed that H2B-Ub altered the binding orientation of hDot1L on nucleosome, which may place the active site of the Dot1L proximal to H3K79. This finding is consistent with the early proposed “corralling” mechanism, in which Ub locates on the surface of the nucleosome to block the unproductive hDot1L binding direction.

### 4.4 New concept probes

#### 4.4.1 Cascade probes

**Fig. 13:**
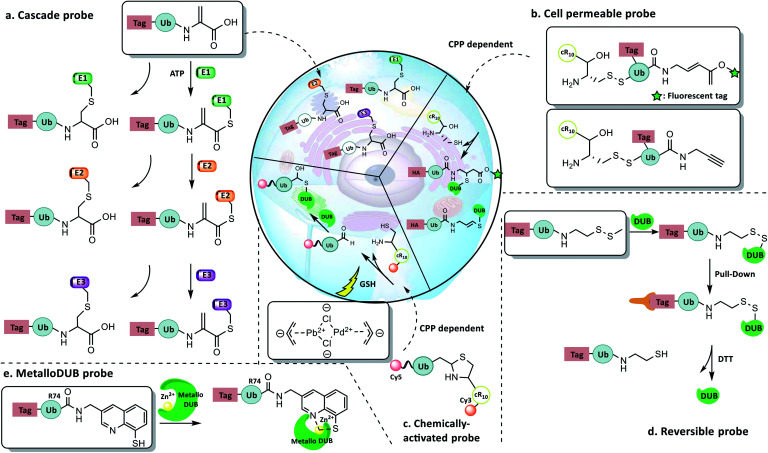
New concept probes. (a) Ub-Dha enzyme cascade probe. (b) Cys-Ub-Prg and -Cys-Ub-VME transmembrane probes. (c) Chemically activated probe. (d) Recyclable probe with a warhead containing a methyl disulfide group. (e) MetalloDUB probe.

Ovaa *et al.* developed an Ub-Dha probe to capture E1, E2, and E3 (ref. 124) (Fig. 13a). The Ub-Dha probe can react with E1 to produce two products: one bearing thioesters that can continue to react with E2 and HECT/RBR E3, and another bearing a dead-end thioether. Using this probe, a variety of E1, E2, and E3 were captured in HeLa and MelJuSo cell lysates. The fluorescently labelled Ub-Dha probe could be further utilized in monitoring the localization and activity of the enzymes *in vivo*. For example, Cy5-Ub-Dha was introduced into cells by electroporation and was found to co-localize with the E2/E3 enzyme BIRC6 on the cytokinetic bridge at the late stage of cell mitosis and also co-localize with the E2 UBE2J1 on the endoplasmic reticulum.

#### 4.4.2 *In situ* probes

Zhuang *et al.* reported cell permeable DUB probes carrying a cell-penetrating peptide (CPP) of either the TAT sequence or cyclic polyarginine cR_10_ (ref. 125) (Fig. 13b). Cell penetrating assays showed the cR_10_ probe had a better cellular uptake compared to TAT attachment. Thus HA-cR_10_-Ub-PA was used for *in situ* proteomic profiling of DUBs in HeLa cells. In addition to the 10 DUBs that could be captured in cell lysate, the HA-cR_10_-Ub-PA probe captured 17 more DUBs, including OTUD4, USP47 and USP9X. This indicated that the disruption of cellular organization might lead to the deconstruction of protein complexes necessary for DUB activity. In parallel to the study, Ovaa *et al.* reported NextGen Ub-TAT, which was used to identify cellular Ub conjugating enzymes.^126^

#### 4.4.3 Probes with on-demand activation

Brik *et al.* developed a Thz-caged Ubv2.3 aldehyde probe that could be activated by [PdCl(allyl)]_2_ (ref. 127) (Fig. 13c). A cR_10_ group was introduced, rendering the probe cell permeable. *In vitro* assays showed that the probe could be chemically activated and then selectively inhibit USP2a. Furthermore, FRET-based *in vivo* assays showed that the probe was uncaged in cells by treatment with [PdCl(allyl)]_2_. Also, the probe was demonstrated to inhibit USP2a in DU45 cells when treated with the Pd complex. This work provides the first case of delivery and activation of a synthetic protein in cells and opens new opportunities for studying dynamic regulation of ubiquitination in cells.

#### 4.4.4 Reversible probe

Ovaa *et al.* reported a reversible monoUb probe with a warhead containing a disulfide group (Fig. 13d), which could label the active Cys of DUB by disulfide exchange.^128^ DUBs captured by the probe (such as UCH-L3, OTUB2, and USP7) can release the probe and regain their activities under mild reducing conditions. The probe was utilized to capture DUBs in HeLa cell lysate, and most of the captured DUBs can be re-captured by the Ub-PA after being reduced by DTT. The reversibility of the probe made it useful for isolating active DUBs or DUB complexes from natural environment and study their activity *in vitro*.

#### 4.4.5 Metalloproteinase probes

The active site of the JAMM/MPN + family DUBs contains Zn^2+^, where no covalent intermediate is generated. Therefore, the aforementioned probes that target Cys are not applicable for capturing this family of DUBs. Ovaa *et al.* reported a mono-Ub probe (Fig. 13e) with a zinc chelator 8-mercaptoquinoline (8-MQ) linked to the C terminus of Ub.^129^ Inhibition assays showed that the probe can inhibit Rpn11/Rpn8 with an IC_50_ value about 2 μM. When incubated with HeLa cell lysates, the probe captured metalloDUBs POH1, AMSH, and AMSH-LP.

## 5. Summary and perspective

This article reviews the development and application of the state-of-the-art Ub-based chemical probes, highlighting the need for innovative technologies and novel concepts to study the ever-increasingly complex facets of Ub biology. Special attention has been given to the use of chemical probes to capture and monitor the enzymes or Ub interactors previously not targeted by conventional probe designs. Progress in this regard will enable a more in-depth dissection of the many enigmatic aspects of ubiquitination in the cells, and elucidation of the mechanisms behind the complex ubiquitination machinery. Furthermore, it should be pointed out that proteomic identification of trapped proteins by the synthetic and semisynthetic probes is still not easy and has become the bottleneck in the field. Also, chemical probes capable of *in vivo* profiling are also increasingly needed. New Ub-based chemical probes will continue to emerge, inspired both by the innovation and progress of chemical protein synthesis, and the fascinating biology and clinical importance of ubiquitination.

## Conflicts of interest

There are no conflicts to declare.
